# Fixation of femoral neck fracture with femoral neck system: a retrospective cohort study of 43 patients

**DOI:** 10.1186/s12891-023-07113-2

**Published:** 2024-01-02

**Authors:** Jae Youn Yoon, Seong-Eun Byun, Young-Ho Cho

**Affiliations:** 1 Department of Orthopaedic Surgery, Dongguk University Ilsan Hospital, Goyang-si, Republic of Korea; 2 Department of Orthopaedic Surgery, CHA Bundang Medical Center, CHA University, Seongnam-si, Republic of Korea; 3 Department of Orthopaedic Surgery, Daegu Fatima Hospital, 99, Ayang-ro, Dong-gu, Daegu, Republic of Korea

**Keywords:** Hip fracture, Femur neck fracture, Femoral neck system, Internal fixation, Complication

## Abstract

**Backgrounds:**

This study aimed to analyze the clinical outcomes of femoral neck fractures (FNF) in patients treated with a femoral neck system (FNS, DePuy Synthes), which is a recently introduced device.

**Methods:**

This retrospective cohort study of 43 patients who underwent osteosynthesis using FNS for FNF between July 2019 and June 2021 with a minimum follow-up of 6 months. The researchers examined the patients’ demographic factors and radiologically evaluated the fracture type and fixation status, bone union, and postoperative complications.

**Results:**

Of 43 patients, 25 were female, and the patients’ mean age and body mass index were 62.1 years and 22.5 kg/m^2^, respectively. According to the Association of Osteosynthesis/Orthopaedic Trauma Association (AO/OTA) classification, the most common fracture types were 31B1.1 and B1.2 (13 cases each), followed by B2.3, B2.1, and B2.2 (seven, five, and four cases, respectively). Radiological bone union was confirmed in 39 patients (90.7%), and the mean time to union was 3.6 months. Two cases of nonunion, one case of lag screw cut-out, and one case of osteonecrosis were confirmed; all four cases later underwent arthroplasty. The mean time to reoperation was 4.5 months. Meanwhile, five patients underwent implant removal after the bone union, and distal locking screw stripping was noted in three patients. All three patients required metal plate cutting to remove the implants.

**Conclusions:**

Osteosynthesis of FNF using the newly introduced FNS showed favorable clinical outcomes and no specific hardware-related complications were reported during the follow-up. However, attention must be paid to the issue regarding distal locking screw failure during hardware removal.

## Background

Femoral neck fracture (FNF) is the most common type of hip fracture in elderly patients; however, it also occurs in young patients as a result of high-energy trauma [1, 2]. The number of patients with FNF continues to increase, and the number of patients with disabilities due to FNF is expected to increase to 21 million in the near future [3]. Owing to the high morbidity and mortality of FNF, providing appropriate treatment and rehabilitation to these patients is a significant issue in reducing the physical and economic burden of patients and society [4, 5].

Surgical treatment is indicated in most FNFs, and the treatment method (osteosynthesis or replacement arthroplasty) is determined according to the anatomical location and angle of the fracture line, degree of displacement, surgeon’s preference, and general condition of the patient. Arthroplasty is usually preferred in elderly patients with displaced FNFs (Garden stage 3 and 4) to reduce the possibility of reoperation and to promote rapid functional recovery. In younger patients, however, functional requirements are relatively high, and they might experience various complications related to arthroplasty in the future. Therefore, joint preservation surgery is usually considered in young patients [6].

Currently, multiple cannulated screws (MCS) or dynamic hip screws (DHS) are the most commonly used fixation devices for the osteosynthesis of FNFs. Fixation using MCS is less invasive, with less bone removal, less disruption of the blood supply, and good torsional stability [7]. However, limb shortening and implant protrusion are often reported, and due to weak resistance to shear force, its use in Pauwels type 3 fractures is limited. Fixation with DHS has advantages in terms of rigid fixation and higher resistance to bending and shear forces. However, DHS fixation requires extensive surgical exposure, including greater bone stock removal and soft tissue damage. Despite the distinct differences in characteristics between the two instruments, both instruments showed good clinical results [8, 9]. No significant difference in clinical outcomes between the two devices was reported in a recent large-scale multicenter randomized clinical study [3].

A new fixation device, the femoral neck system (FNS; DePuy Synthes, MA, USA), has the combined advantages of the former two devices. Fixation with FNS is minimally invasive similar to MCS fixation because of its small implant footprint and can shorten the operation time and reduce the risk of radiation exposure owing to its simple surgical method [10, 11]. According to biomechanical studies, the overall construct stability of FNS is similar to that of DHS and superior to that of MCS [12]. These biomechanical advantages provide a theoretical basis for using FNF in Pauwels type 3 (vertical shear) FNF [13].

Despite these theoretical and biomechanical advantages, only a few studies report the clinical outcomes of osteosynthesis using FNS, and the results are inconsistent among the studies. Therefore, we aimed to share our experience of using FNS in patients with FNF and perform a failure analysis in several cases. We also discussed the potential problems regarding implant removal, which have never been addressed in previous studies.

## Methods

### Study design and patient selection

This retrospective cohort study was conducted at three different institutions, and the respective institutional review boards approved this study. The requirement for formal informed consent was waived because of the retrospective nature of the study. We reviewed the medical records of 49 patients who underwent osteosynthesis using FNS for mono-traumatic FNF between July 2019 and June 2021, and those who satisfied the minimum follow-up period of 6 months were enrolled in the study. In agreement, osteosynthesis was performed in patients aged < 65 years, even with Garden stage 3 and 4 fractures, unless there were no particular limitations. In patients over 65 years of age, the surgical method was decided considering the patient’s medical condition, fracture displacement, and presence of arthritic changes.

### Data collection

Clinically, we collected patients’ demographic factors, including age, sex, body mass index (BMI), medical history (including a history of diabetes mellitus, hypertension, deep vein thrombosis, pulmonary embolism, stroke, chronic kidney disease, and liver cirrhosis), and medications (including anticoagulants or antiplatelet agents). The Koval’s grade and Charlson comorbidity index (CCI) were used to assess the individual preoperative ambulation state and medical comorbidities. The operation time, type of anesthesia, length of hospital stay, and complications during admission were also reviewed.

Radiologically, the initial fracture pattern, reduction status, implant position, tip-apex distance (TAD), bolt sliding length, time to union, and any other complications (such as osteonecrosis, nonunion, infection, and post-traumatic arthritis) were also reviewed. The Garden, Pauwels, and AO/OTA classifications were used to distinguish the fracture types, and the Cleveland index was used to analyze the location of the bolt [14, 15]. TAD and bolt sliding length were calculated using the ratio of the radiographic measurements [16]. Delayed union (4 months after fixation) and nonunion (6 months after fixation) were determined when the patient complained of persistent pain that did not improve gradually, and radiological improvement (callus formation and cortical bone bridging in three different directions on both anteroposterior and translateral views) was not observed. In these patients, three-dimensional computed tomography was performed to evaluate surgical failure and plan for revision surgery.

### Surgical procedures and postoperative rehabilitation

Each surgeon at a different institution performed the surgeries. The patients were placed in a supine position on a fracture table with a conventional traction device, and fluoroscopy was used to aid the operation. A closed reduction technique with minimal skin incision (4–5 cm) was routinely performed; however, mini-open reduction with Hoffman retractor or Kelly forceps was necessary in several cases, which required accurate fracture reduction. Patients were trained to start protected weight bearing (30–50% of the individual body weight) using an assistive device (walker or double crutches) 1 or 2 days after the surgery. Patients were discharged from the hospital when independent ambulation with an orthosis was possible and the pain was controlled.

After discharge, the patients were regularly followed up at the outpatient clinic at 6, 12, and 36 weeks postoperatively. The outpatient schedule was adjusted according to the situation if any changes in the patient’s medical condition or surgery-related complications were observed or suspected. Patients were educated on partial weight bearing using an assistive device (walker or double crutches) starting 1 or 2 days postoperatively for up to 6 weeks. Following that, tolerable weight bearing was recommended for an additional 6 weeks when the clinical bone union was mainly observed.

### Statistical analyses

Metric data are presented as the mean values with a 95% confidence interval (CI), and categorical variables are presented as absolute frequencies and percentage distributions. Student’s t-test or Mann–Whitney test was used for metric data, and chi-square test or Fisher’s exact test was used for categorical data. Univariate logistic regression analysis was used to assess the risk factors associated with revision surgery. All statistical analyses were performed using SPSS statistical software version 21 (IBM Co., Armonk, NY, USA), and a statistical significance was set at *p* < 0.05.

## Results

### Demographics of the patient

A total of 49 patients underwent surgical treatment using FNS during the study period; among them, 43 patients satisfied the inclusion criteria and were included in the analysis. The mean age of the patients was 62.1 (range: 57.7–66.5) years, and 25 (56%) patients were female. The mean follow-up duration of the enrolled patients was 12.5 months (range: 11.1–14.0). Additional patient demographic factors and detailed fracture classifications are presented in Table 1. According to the Garden classification, 31 (72.1%) patients had stable fractures and 12 (27.9%) had unstable fractures. According to the Pauwels classification, 33 (46.7%) patients had a fracture angle of less than 50 degrees (Pauwels type 1 and 2), and 10 (23.3%) patients had a vertical shear fracture (Pauwels type 3).

**Table 1:** Patients’ characteristics

Demographic factors	mean (95% CI range)
Age (years)	62.1 (57.7–66.5)
Sex ratio (Female : Male, n)	25:18
Body Mass Index (kg/m^2^)	22.6 (21.7–23.5)
Affected side (Right : Left, n)	27:16
Preoperative Koval grade	1.4 (1.1–1.6)
Charlson Comorbidity index	3.0 (2.4–3.6)
Follow-up duration (months)	12.5 (11.1–14.0)
**AO/OTA Classification (31B, femoral neck)**	**n (%)**
**B1 (Subcapital)**	
B1.1 (valgus impacted)	13 (30.2%)
B1.2 (nondisplaced)	13 (30.2%)
B1.3 (displaced)	0
**B2 (Transcervical)**	
B2.1 (Simple)	5 (11.6%)
B2.2 (Multifragmentary)	4 (9.3%)
B2.3 (Shear)	7 (16.3%)
**B3 (Basicervical)**	
B3 (basicervical)	1 (2.3%)
**Garden Classification**	**n (%)**
Garden 1 (incomplete, valgus impacted)	15
Garden 2 (complete, undisplaced)	16
Garden 3 (complete, partially displaced)	4
Garden 4 (complete, completely displaced)	8
**Pauwels Classification**	**n (%)**
Pauwels 1 (< 30 degrees)	8
Pauwels 2 (30–50 degrees)	25
Pauwels 3 (≥ 50 degrees)	10

* Numeric data are presented in mean value (95% confidence interval range)

### Surgical factors

**Fig. 1:**
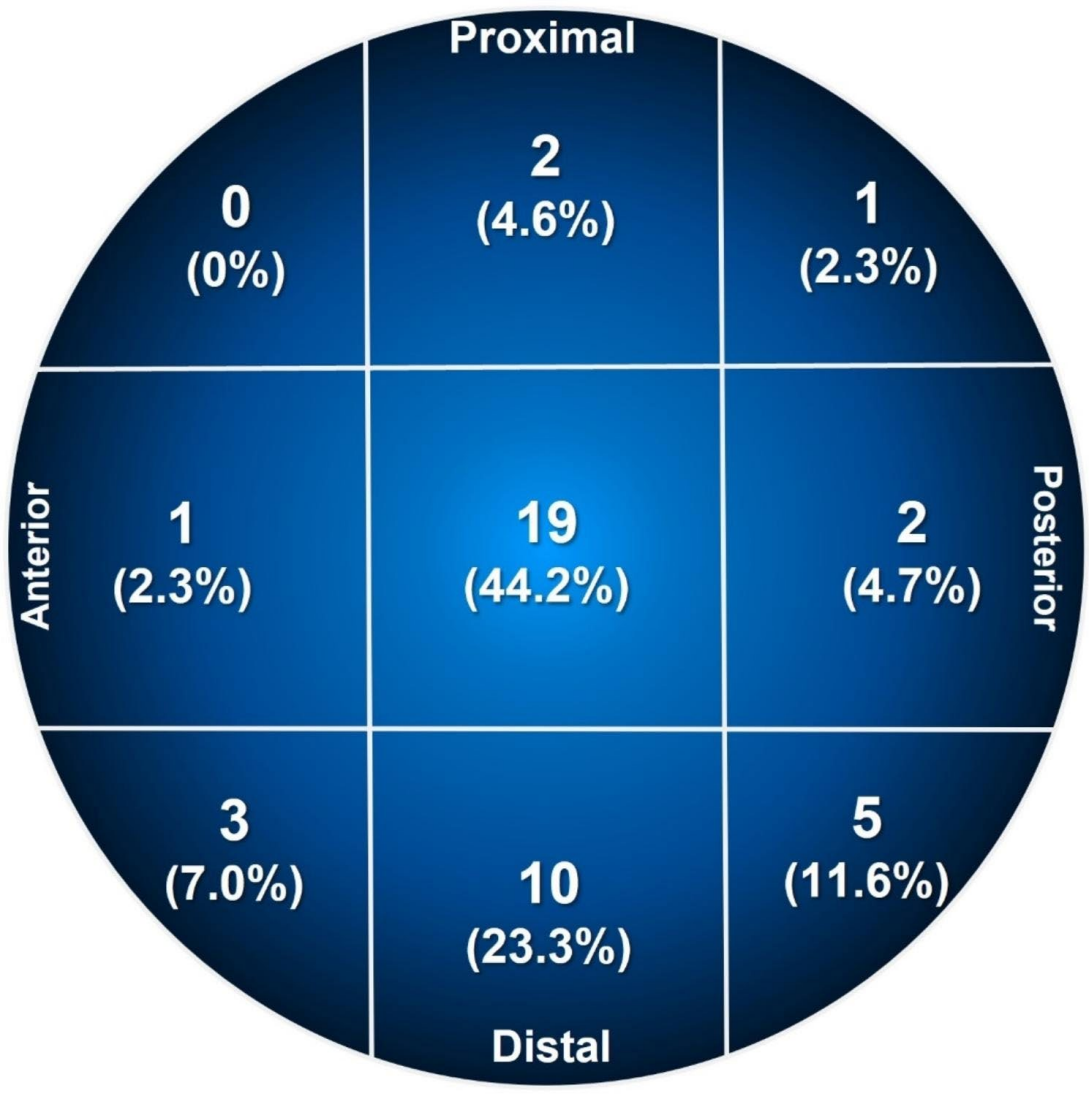
Illustration showing the location of the bolt in the femoral head using a femoral neck system (Cleveland index)

The mean duration from the time of injury to surgery was 1.6 (range: 1.1–2.1) days, and the mean operation time (incision to closure) was 47.9 (range: 43.8–51.9) minutes. Thirty-six (84%) patients underwent general anesthesia and seven (16%) underwent spinal anesthesia. We used a one-hole plate for all patients, except one. Bolt and anti-rotation screws were fixed at the same length in 32 (74.4%) patients, as initially recommended by the manufacturer. An anti-rotation screw 5 mm longer than the bolt was used in 11 (25.6%) patients to obtain stronger rotation resistance. When the bolt position was measured radiographically, the average TAD was 21.1 (range: 18.5–23.8) mm, and 39 (88.6%) patients satisfied the TAD of a bolt within 25 mm. The insertion position of the bolt in the femoral head was diagrammed using the Cleveland index (Fig. 1). The center-center and center-inferior positions were used in 19 (44.2%) and eight (23.3%) cases, respectively. Insertion of bolts in the anterior and superior positions was avoided.

### Outcomes

No acute perioperative complications were observed during admission, except in two cases that showed short-term postoperative delirium. However, the delirium subsided within 3 days, and both patients were successfully discharged from the hospital after rehabilitation. No postoperative infections were noted during the follow-up period.

### Complications

**Fig. 2:**
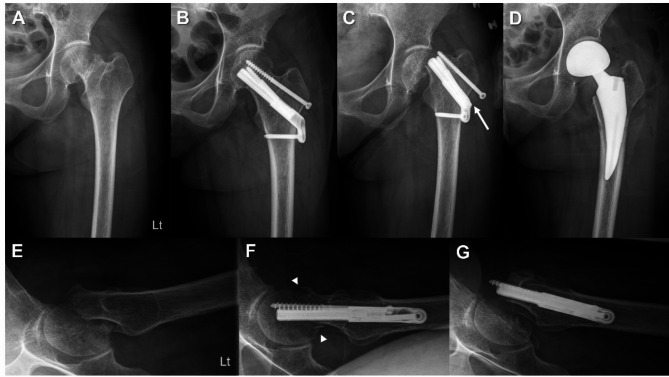
Case of a 48-year-old female femoral neck fracture (FNF) patient with underlying moderate-grade Intellectual disability. (**A, E**) The patient was diagnosed with Garden type 4 FNF, and the bone quality was poor due to general medical conditions. (**B, F**) In Immediate postoperative x-rays, the position of the bolt was not central, and posterior angulation of the femoral head was noted. We inserted an additional screw to maintain the reduction status and to compensate for the rotation instability. (**C, G**) Fixation failure occurred 1.5 months after the operation. The bolt slid laterally to a maximum degree (20 mm), and the additional screw was also pulled out. (**D**) We performed hemiarthroplasty after implant removal at 1.6 months after the initial surgery

Four (9.1%) patients complained of residual pain in the affected hip at the final follow-up, and six (13.6%) presented with limping gaits. Regarding serious complications during follow-up, two cases of nonunion, one case of screw cut-out, and one case of post-traumatic osteoarthritis (due to osteonecrosis) were observed. All four patients later underwent arthroplasty due to complications (Fig. 2). The detailed data of the patients who experienced surgical failure are presented in Table 2. When univariate regression analysis was performed to determine the risk factors for revision surgery, prior stroke history (*p* = 0.018, odds ratio [OR] = 18.5), longer TAD (*p* = 0.016, OR = 1.24), and longer bolt sliding length (*p* = 0.015, OR = 2.02) showed statistically significant results (Table 3). However, the multivariate analysis failed to derive statistically significant results for these variables.

**Table 2:** Detailed data of the patients with surgical failure

Case	Age	Sex	BMI	Side	Koval grade	Stroke history	CCI	AO/OTA	Garden	Pauwels	TAD (mm)	Cleveland Index	Bolt sliding (mm)	Cause of revision	Time to revision (months)	Type of revision
#10	48	F	22.2	Left	1	No	1	B2.1	4	2	30.5	8	13.5	nonunion	1.6	BPHA
#13	59	M	24.0	Right	1	Yes	3	B2.2	3	3	37.8	9	8.4	nonunion	7.2	BPHA
#14	55	M	20.1	Left	1	Yes	3	B2.1	4	2	59	3	12.3	bolt cutout	1.7	THA
#33	78	M	25.7	Right	2	No	8	B1.2	2	2	24.9	5	13.2	Osteonecrosis	7.5	THA

BMI, body mass index; CCI, Charlson comorbidity index; TAD, tip-apex distance; THA. total hip arthroplasty

**Table 3:** Risk factors associated with reoperation in FNS

Variables	No Reoperation (n = 39)	Reoperation (n = 4)	*p* value	Odds ratio	95% CI
Age at surgery (years)	62.3 (57.7–67.0)	60.0 (54.9–64.1)	0.761	0.989	0.921–1.062
Sex			0.191	4.8	0.456–50.498
Female	24 (61.5%)	1 (25%)			
Male	15 (38.5%)	3 (75%)			
BMI (kg/m^2^)	22.6 (57.7–67.0)	23.0 (21.2–24.8)	0.799	1.047	0.737–1.488
Stroke history	2 (5.1%)	2 (50%)	0.018	18.5	1.642–84.62
Charlson comorbidity index	3.4 (2.6–4.3)	3.8 (2.7–4.8)	0.982		
Garden classification			0.058	0.100	0.009–1.083
Garden 1 & 2 (Stable)	30 (76.9%)	1 (25%)			
Garden 3 & 4 (Unstable)	9 (23.1%)	3 (75%)			
Pauwels classifications			0.886		
Pauwels 1	8 (20.5%)	0			
Pauwels 2	22 (56.4%)	3 (75%)			
Pauwels 3	9 (23.1%)	1 (25%)			
Tip-apex distance (mm)	19.4 (17.6–21.2)	38.1 (24.4–51.7)	0.016	1.244	1.041–1.0485
Cleveland index			0.444	0.444	0.056–3.538
Ideal (zone 5, 8)	27 (69.2%)	2 (50%)			
Non-ideal (zone 4, 6, 7, 9)	12 (30.8%)	2 (50%)			
Bolt sliding length (mm)	2.6 (1.7–3.4)	11.9 (9.4–14.3)	0.015	2.017	1.147–3.545

**Fig. 3:**
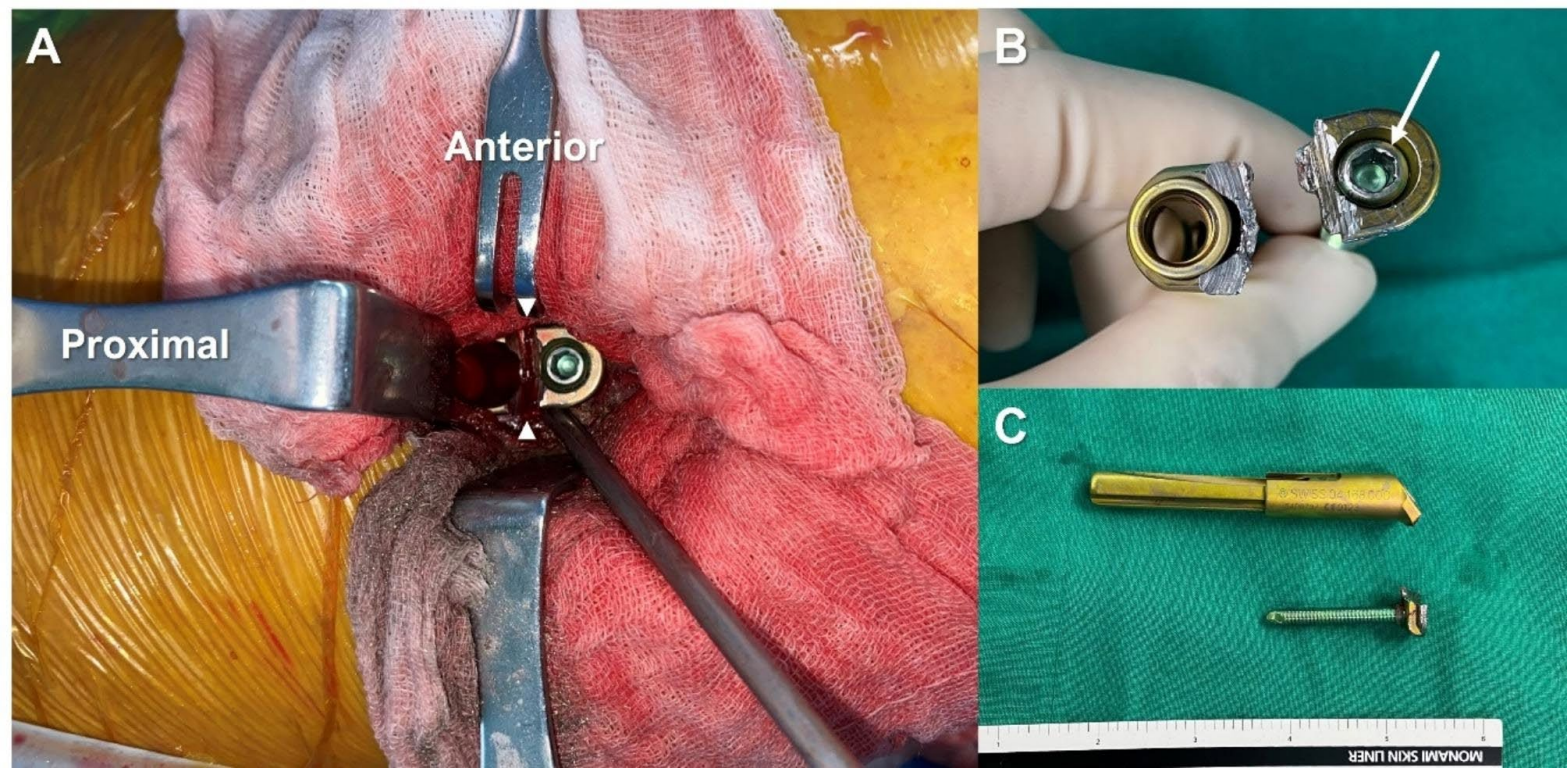
Case of failure in implant removal of a 61-year-old female patient. (**A**) Due to a jammed locking screw, the plate had to be cut in half using a metal cutting saw (arrowheads) to get it removed from the patient. (**B**) Stripping of the internal hexagon in the head of a locking screw (arrow) occurred easily. (**C**) A gross photo was taken after complete implant removal. The screw and the remaining plate were easily pulled out together, and no screw shank fracture occurred

After the complete bone union was confirmed, five patients (mean age, 43.8 years; mean BMI, 23.1 kg/m^2^; female:male ratio = 2:3) underwent additional surgery for implant removal, and the mean duration between initial surgery and implant removal was 17.6 (range: 14.7–18.9) months. During the implant removal procedure, distal locking screw stripping occurred in three patients. The implant could only be removed after forced metal plate cutting with a Hercules plate cutter or saw due to screw breakage, but implants were successfully removed from all the patients (Fig. 3).

## Discussion

The overall failure rate of FNS in our study, with a mean follow-up of 12.5 months, was 9.3% (4/43 patients), and the results were similar or superior to those reported in previous studies (8.8–21%) [3, 17, 18]. Even in patients with Pauwels type 3 fracture, which is a relative contraindication to MCS fixation, complete bone union was achieved without major complications such as nonunion or angular deformation in most patients (Table 1). With regard to the favorable clinical outcome of FNS, Stoffel et al. [12] demonstrated the biomechanical strength of FNS compared to the existing MCS and DHS through a biomechanical study conducted in 2017. According to their study, it was confirmed that the FNS had a higher axial stiffness and better resistance to varus tilting and construct failure compared to MCS and DHS. A lower incidence of femoral neck and leg shortening was observed with FNS than that observed with MCS. Despite the minimally invasive mechanical properties, the biomechanical stiffness was similar to that of DHS. Recently, Moon et al. [13] reported that FNS can provide stronger structural stability than DHS in displaced basicervical neck fractures, which are considered rotationally unstable. Since our study included data on patients with all types of FNFs within a certain period for which osteosynthesis was planned, our data would be insufficient to statistically prove the clinical superiority of FNS in certain criteria or specific fracture patterns. However, based on the favorable clinical results, although limited, we believe that FNS can be used easily and safely for various types of FNFs.

The major postoperative complications included fracture nonunion, osteonecrosis, and screw cut-out. The nonunion rate reported in previous studies varied from 6 to 33%, and the rate in our study was much lower (4.7%, 2/44 patients) [19–21]. Similarly, post-traumatic osteonecrosis was reported in only one case (2.3%) during the follow-up period, and the result was also significantly lower than that reported in previous studies (4.5–11%) [3, 22]. Most cases of fracture nonunion are diagnosed within 10–12 months, and given our mean follow-up period was 12.5 months, the favorable outcome might be attributable to the superior biomechanical stability of the FNS. In contrast, osteonecrosis may be detected even 2 years or more after trauma. Since we did not routinely use more invasive imaging modalities (such as magnetic resonance imaging and computed tomography) in addition to simple radiographs, the actual incidence of osteonecrosis might have been overlooked.

Although the clinical significance of surgical timing is controversial, multiple factors such as patients’ sex, BMI, age, fracture type, time elapsed from injury to surgery, and the quality of reduction are known to affect the outcomes and risk of reoperation [3, 18, 20, 23]. Stassen et al. [18] reported that the patient’s age and presence of chronic lung disease were closely related to reoperation. Meanwhile, Davidson et al. [17] revealed that the patient’s age, surgeons’ seniority, and proper placement of FNS were closely associated with reoperation. Or results (Table 3) found that prior stroke history, TAD, and bolt sliding length were related to reoperation. It was confirmed that both case identification numbers 13 and 14, presented in Table 2, had a prior history of stroke. Despite the history of stroke, the pre-injury ambulatory function of the patients was not severely limited (Koval’s grade 1 and 3), and the CCI score, which indicates the degree of patients’ morbidity, showed no difference compared to the group of patients that did not undergo revision surgery. Therefore, clinical significance of patients’ stroke history should be validated through further detailed analysis.

Radiologically measured TAD and bolt sliding length were also the major risk factors for reoperations. First, the TAD exceeded 25 mm in three out of four patients who underwent reoperation, and the TAD in the other case was 24.9 mm, which was also near the 25 mm value. Only a few studies have studied the relationship between FNS and TAD, and the results have been inconsistent. Jung et al. [24] reported that the length and position of the bolts play an important role in obtaining biomechanical stability in Pauwels type 3 fractures. However, Stassen et al. and Davidson et al. [17, 18] did not observe a statistically significant association between TAD and revision surgery. Nevertheless, both authors emphasized that the precise central location of the bolt is of substantial importance in surgical prognosis. The bolt length is provided in 5 mm increments; therefore, some difficulties in finely adjusting the TAD exist. However, it is advisable to keep the TAD of the bolt within 25 mm and insert it in a position as central as possible. Meanwhile, bolt sliding is a process that occurs acutely during intraoperative inter-device compression and gradually during the natural healing process. The bolt allowed for sliding on the angular plate up to a maximum length of 20 mm. In our study, in the patients that underwent revision surgery, however, the degree of sliding was longer than the mean value and occurred very rapidly, especially in patients with bolt cut-out and nonunion due to reduction failure. Therefore, verifying the causal relationship may be limited despite a statistically significant correlation.

Finally, locking screw stripping occurred in 60% of the cases (3/5 cases) during implant removal after the complete bone union. With regard to the reason for hardware removal, three out of five patients complained of postoperative pain at the surgical site. When a physical examination was performed at an outpatient clinic, positive direct tenderness was confirmed by the physicians. In the remaining two cases, surgery was performed due to the patient’s personal request. One young business worker had a foreign body sensation and had inconvenience related to security check-out screening at overseas airports. The other was a professional bicyclist with concerns about future periprosthetic fractures. This has not been reported in previous studies and various mechanisms have been considered to explain this phenomenon.

The footprint of the plate in contact with the lateral femoral cortex, which secures angular stability, is small, and only one or two 5.0 titanium locking screws support the load. The excessive stress applied to the thread between the plate and screw head may result in mechanical locking or jamming of the threaded head of the screw, which may cause shear failure of the screw head. Another hypothesis is that the location of the locking screw insertion may also be related to this phenomenon. The distal locking screw secured the plate by obtaining a bicortical fixation at the level of the lesser trochanter of the proximal femur. In this area, the cortical bone is very thick, and the calcar femorale is located at the posteromedial junction of the femoral neck and diaphysis, making the actual working length of the thread much longer. Therefore, the torque used for screw removal exceeded the strength of the thread, and the thread pattern was destroyed during the removal process, making it impossible to release the thread.

Since implant removal is primarily considered in active young patients, the good bone quality and thick cortical bone in these patients may act as obstacles to implant removal. To avoid such unexpected difficulties during surgery, it is necessary to prepare any available devices, such as a reverse-threaded screw extractor set, diamond burr, or trephine drill. In some cases, a plate cutting tool may be helpful (Fig. 3). Meticulous attention must be paid not to spread metal debris that is inevitably generated during this undesired procedure. Moreover, notifying the patients before the surgery is critically important.

Our study is limited by its non-randomized design, lack of patient-reported outcome measures, and a relatively small number of participants. However, this study is significant because the main purpose of this study was to discuss the initial experience of using a new fixation device (FNS) and evaluate the radiological complications, such as nonunion, osteonecrosis, and screw cut-out. Furthermore, despite the small number of study participants, we identified several statistically significant factors related to surgical failure.

## Conclusion

Based on our clinical experience with a mean follow-up of 12.5 months and other recent studies, we believe that the FNS can be safely and easily used for various types of FNFs. However, future large-scale randomized controlled studies are required to validate mid- to long-term clinical outcomes between DHS and/or MCS with FNS, as well as post-market surveillance regarding implant failure and screw stripping.

## Data Availability

The datasets used and analyzed during the current study are available from the corresponding author, as a supplementary file, on reasonable request.
